# Is Mutans Streptococci count a risk predictor of Early Childhood Caries? A systematic review and meta-analysis

**DOI:** 10.1186/s12903-023-03346-8

**Published:** 2023-09-07

**Authors:** Sheetal Manchanda, Divesh Sardana, Simin Peng, Edward C. M. Lo, Neeta Chandwani, Cynthia K. Y. Yiu

**Affiliations:** 1 Department of Paediatric Dentistry, Faculty of Dentistry, The University of Hong Kong, Hong Kong S.A.R, Hong Kong; 2 Department of Paediatric Dentistry, Health Sciences Centre College of Dentistry, The University of Oklahoma, Oklahoma, USA; 3 Department of Dental Public Health, Faculty of Dentistry, The University of Hong Kong, Hong Kong S.A.R, Hong Kong; 4 Department of Developmental Biology, Harvard School of Dental Medicine, Boston, MA USA

**Keywords:** Streptococcus mutans, Dental caries, Risk, Child, Preschool

## Abstract

**Background:**

The review aims to determine the risk predictability of mutans streptococci in the development of carious lesions in children with primary dentition.

**Methods:**

Longitudinal observational studies with at least 6 months follow-up and evaluating mutans streptococci presence in caries-free children under 6 years of age for the development of any cavitated or non-cavitated carious lesion. Six databases and grey literature were searched without any restrictions. Risk of bias was evaluated using the New Castle Ottawa scale for longitudinal studies, and the certainty of the evidence was evaluated by Grading of Recommendations Assessment, Development and Evaluation using GRADEpro software. Meta-analysis was performed using a random effect (DerSimonian and Laird, DL) model, and heterogeneity was evaluated using tau-squared, I^2^ statistics and prediction interval. Sensitivity analysis was performed to assess the relationship between the mutans streptococci presence at baseline and the caries development, according to the sample and methods used for the microbiological assessment and the length of follow-up of the studies. Publication bias was checked by funnel plot using a random effect (DerSimonian and Laird, DL) model.

**Results:**

Twelve studies met the inclusion criteria and were included in the review. Four studies received a maximum of 9 stars, and among the remaining eight studies, six received 8 stars and the rest two studies were assigned 7 stars in the risk of bias scale. After pooling the results quantitatively, odds ratio (OR) was found to be 4.13 (95% CI: 3.33, 5.12), suggesting that children with mutans streptococci had 4 times higher odds of developing caries later (*p* < 0.001). Four studies were pooled to compare future caries experience among children with and without mutans streptococci at baseline, obtaining standardized mean difference (SMD) of 0.85 (95% CI: 0.33, 1.37), indicating a large effect (*p* < 0.001). Certainty of evidence was found to be moderate, and no publication bias was reported by the funnel plot criteria of symmetry.

**Conclusions:**

Presence of mutans streptococci in a preschool child is a risk predictor for future caries experience. Early identification of children with increased caries-risk may facilitate in implementation of appropriate preventive strategies.

**Supplementary Information:**

The online version contains supplementary material available at 10.1186/s12903-023-03346-8.

## Introduction

Dental caries is a potentially infectious and transmissible disease with a reported high global prevalence of 46.2% in the primary teeth (95% CI: 41.6–50.8%) [1]. Early Childhood Caries (ECC) is defined as the presence of one or more carious (non-cavitated or cavitated lesions), missing (due to caries), or restored dental surfaces in any primary tooth in a child younger than 6 years [2]. Despite the implementation of various prophylactic preventive programmes focussing on fluoride therapy and dental education, the prevalence of ECC among 1, 2, 3, 4, and 5 years old children remains a cause of concern with values of 17%, 36%, 43%, 55% and 63%, respectively [3].

Various biological, physical, behavioural, environmental and lifestyle-related risk indicators have been found to be associated with ECC and among them, microbiological factors have been well recognized [4, 5]. Mutans streptococci infection has been considered to be associated with the caries process due to its acidogenic and aciduric properties and the production of specific intra- and extracellular polysaccharides, which promotes its adhesion on the tooth surface [6]. Furthermore, studies [7, 8] have investigated the mutans streptococci count and its genetic diversity in various oral cavity sites in preschool children with different caries statuses and found that its count differs according to the samples used and the child's caries status.

Numerous methods have been employed by various studies for the identification and quantification of microorganisms in oral samples, which may include microscopy, culture methods, biochemical techniques and species-specific DNA probes. Besides being time-consuming and labour-intensive, traditionally-used culturing techniques for detecting bacteria on selective media limit the detection of all microorganisms involved in the caries process. Moreover, molecular methods have been introduced recently for microbial quantification based on variations of DNA analysis like amplification [Polymerase Chain Reaction (PCR), real-time PCR] or sequencing-based employing specific primer sequences for identifying a particular bacterial DNA through rapid amplification [9].

Clinical studies have been conducted to quantify the association between the primary cariogenic microorganism, mutans streptococci, with caries in the deciduous dentition; however, despite considerable research, the question about mutans streptococci count being a risk predictor of ECC is limited by the study designs (e.g. cross-sectional or case–control). Moreover, no previous evidence-based summary has been generated from longitudinal studies that provide a comprehensive approach to answering the research question. Therefore, the present systematic review was performed aiming to detect the risk predictability of mutans streptococci with the longitudinal development of carious lesions in children with primary dentition.

## Methods

### Protocol and registration

The current review methodology was formatted according to the guidelines suggested by the Cochrane Handbook for Systematic Reviews [10] and is reported per the PRISMA (Preferred Reporting Items of Systematic Reviews and Meta-analysis) statement and checklist [11]. The protocol was registered *a priory* in the PROSPERO database (Registration number- CRD42022349922).

### Eligibility criteria

#### Inclusion criteria

Type of studies: Only longitudinal observational studies were included that assessed the presence of mutans streptococci in oral samples at the beginning of the study in children less than 6 years of age and who were followed for at least 6 months for the longitudinal development of caries in the primary dentition. The detailed PECO (Population, Exposure, Control and Outcome) schema followed is below:

- Population*:* Children younger than 6 years of age, with no evidence of any cavitated or non-cavitated lesions at recruitment.
- Exposure: Presence of mutans streptococci in the sample.
- Control: Absence of mutans streptococci in the sample.
- Outcome: The proportion of children developing any cavitated or non-cavitated carious lesion in the primary dentition during follow-up with/without decayed, missing, and filled index at tooth or surface level (dmft/dmfs).

#### Exclusion criteria

Studies in animals, or children with special needs, or undergoing orthodontic treatment were excluded. Narrative reviews, systematic reviews, case reports, conference reports, case–control, cross-sectional studies and case series were excluded from the present systematic review. Studies with any kind of intervention like fluoride, restoration, sealants, education were excluded.

Some studies recruited caries-free children along with those who had caries at baseline; however, we extracted data for only caries-free children who were followed to investigate the development of caries. Studies missing the data for caries-free children or the mutans streptococci presence at baseline, were excluded.

### Information sources and literature search

A systematic search of the literature was performed using broad MeSH terms and keywords on 15^th^ Nov 2022 by two investigators (SM and DS) with no start date or language restrictions. Six databases searched were: Medline (via Ovid); Embase (via Ovid); Scopus; PubMed; CINAHL; and Web of Science. After removing duplicates, the titles and abstracts were screened against the predetermined eligibility criteria to decide upon the inclusion for further full-text reading. The record was subjected to full-text reading if the abstract provided an unequivocal interpretation regarding inclusion or exclusion. The reference lists of the included articles and previous published systematic reviews were hand-searched for any potentially relevant article which could be included. To identify grey literature, www.opengrey.eu and Google Scholar were also searched for any unpublished material or dissertations.

### Study selection

The results obtained through the searching of the electronic databases, journals, and grey literature were systematically managed using Endnote X 8.2 software for Windows (Clarivate Analytics, Philadelphia, USA) but was exported into the Covidence (Covidence systematic review software, Veritas Health Innovation, Melbourne, Australia) available at www.covidence.org). Two independent investigators (SM and DS) scanned the titles and their respective abstracts according to the set inclusion and exclusion criteria after the removal of duplicates. Articles that did not provide detailed methodology underwent full-text reading. A third investigator (CKY) was consulted in case of any discrepancy between the two investigators. Cohen's kappa coefficient (**κ**) was calculated to establish the level of interrater agreement among two reviewers after full-text reading [12].

### Data extraction

Two authors (SM and DS) independently extracted the following data from the included studies on a piloted data extraction sheet: author (year of publication), study location, sample size, participants' age distribution, study design, caries diagnostic criteria, study duration, oral samples used for microbial assessment, the method used for mutans streptococci count assessment, result and source of funding. A 2 × 2 table was constructed for performing meta-analysis and for reporting the results of the included studies.

### Risk of bias in individual studies

The New Castle Ottawa scale [13] for longitudinal studies was used by two authors (SM and DS) independently to assess the methodological quality of the included studies. The scale consisted of 8 questions dispersed across the three domains: the selection (4 questions), the comparability (1 question), and the outcome of interest for cohort studies (3 questions). The maximum score for each question was 1 star, except for the question on comparability, which could be assigned 2 stars; therefore, the highest possible score for the scale was 9 stars. Any disagreements over the bias assessment were resolved if necessary, by discussion and consensus with the third reviewer (CKY).

### Summary measures and quantitative synthesis

Statistical heterogeneity of the included studies was evaluated using tau-squared and I^2^ measurement with significance indicated by *p* < 0.05. Meta-analysis was planned as defined in the registered protocol. A random effect (DerSimonian and Laird, DL) meta-analysis model was performed to compute the Odds Ratio (OR) for binary variables (presence or absence of caries), and the continuous variables (mean number of mutans streptococci between baseline and follow-up), the difference in the means was calculated using inverse variance method. Stata version 16 (StataCorp, College Station, TX, USA) was used to perform all the analyses at an alpha of 0.05 and 95% confidence intervals.

### Additional analysis

Additional analysis included a sensitivity analysis that was performed to identify the relationship between the presence of mutans streptococci at baseline and the longitudinal development of caries according to the sample (plaque or saliva) and method (semi-quantitative using Dentocult/ other commercially available kits or quantitative based on the culturing/ molecular techniques) used for the microbiological assessment. Another sensitivity analysis was performed according to the length of follow-up of the studies.

### Publication bias

To assess the publication bias, funnel plot technique and Eggers test were planned if more than 10 studies were included in the analysis for a valid inference from the results using a random effect (DerSimonian and Laird, DL) model. The criterion of symmetry was used to inspect visually the plots generated.

### Certainty assessment

The certainty of the evidence was identified by the Grading of Recommendations Assessment Development and Evaluation (GRADE) approach [14, 15] using GRADEpro software (http://gradepro.org/) independently by two authors (SM and DS) at four levels: high, moderate, low and very low. Discrepancy, if any, was sorted out through discussion with a third author (CKY).

## Results

### Study selection

**Fig. 1:**
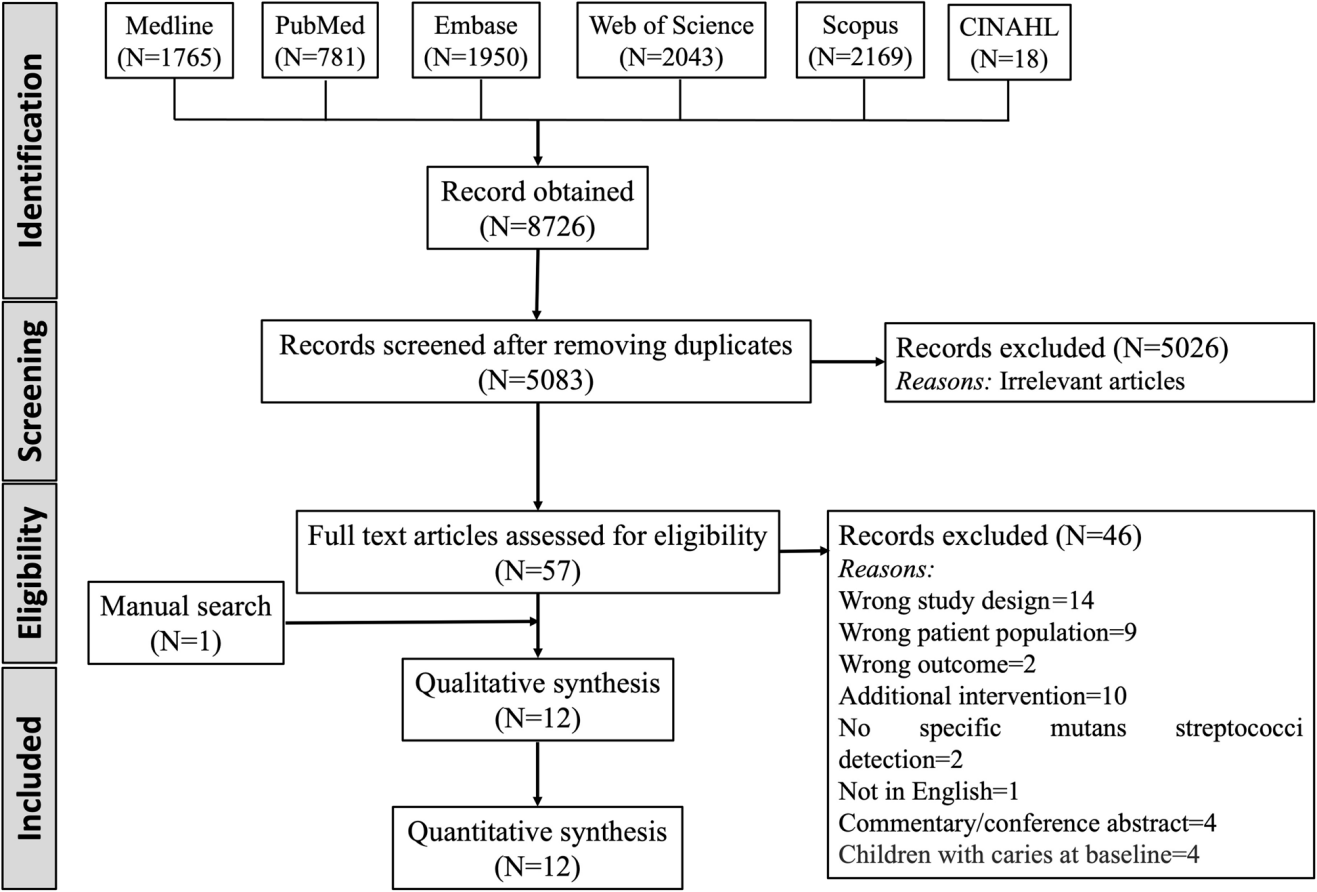
PRISMA flow diagram for the study selection

The study selection process is presented in the PRISMA flow diagram in Fig. 1. Searching databases yielded 8726 records which were imported into Covidence. After removing duplicates 5083 articles were screened for titles and abstracts, retrieving 57 articles potentially eligible for full-text reading. A total of 11 articles were found after full-text reading for qualitative analysis. One article [16] was identified through a manual search satisfying the inclusion criteria and was included in the review. Thus, in total 12 studies were included in the review. The complete search strategy of the electronic databases searched with the yields (number of hits) is presented in the Supplementary Table 1. The κ value for the selection of the studies from full-text articles was 0.86, indicating a substantial level of agreement. The reason for the exclusion of articles after full-text reading is described in Supplementary Table 2.

### Characteristics of the included studies

The summary of the studies included in the review is presented in Supplementary Table 3.

Four studies [17–20] were conducted in Japan, three [21–23] in Finland, two each in USA [24, 25] and Sweden [16, 26] and one in Singapore [27]. The studies were commenced on a total of 3313 children that were caries-free at the start of the study, with the follow-up duration varying from 6 months in two studies [17, 18], 1 year in four studies [19, 24, 26, 27], and 2 years in four studies [25, 16, 20, 23]. Also, two studies had a follow-up of 31 months (median) [21] and 42 months [22] respectively.

The samples used for the microbial assessment varied among the studies. Two studies [21, 22] used plaque samples for detecting the presence of mutans streptococci, while saliva samples were used by 7 studies, among which five [16, 19, 24–26], collected unstimulated saliva samples, while two studies [20, 27] collected stimulated saliva after paraffin wax chewing for 1 min. Additionally, 2 studies [17, 18] used both plaque and saliva samples for mutans streptococci quantification*.* However, only one study [23] reported to have used both plaque and saliva samples, but the data of only plaque samples were presented and thus, included in the analysis.

Quantitative method of bacterial identification was used in 5 studies [19, 21, 23–25] by CFU counting after culturing on Mitis Salivarius bacitracin agar (MSA). However, one study [16] assessed the caries risk depending on the presence or absence of growth of mutans streptococci in bacterial cultures. Six studies [17, 18, 20, 22, 26, 27] used semi-quantitative methods via commercially available kits like Dentocult-SM Strip mutans (D-SM; Vivadent, Schaan, Liechtenstein).

Caries diagnosis criteria also varied among the included studies five studies using World Health Organization (WHO) criteria [17–20, 27], two studies [16, 26] used Koch (1967) criteria [28], two studies [24, 25] used Radike (1968) criteria [29], one study [23] used Moller's (1966) clinical criteria [30]. Two studies [21, 22] reported only using Decayed-missing-filled index at surface or tooth level for caries assessment. Radiographs were used for proximal clinical examination only in one study [16]. The presence of initial non-cavitated carious lesions, also called white spot lesions (WSLs), was diagnosed clinically in only two studies [16, 26].

The funding support used by the included studies for conducting the research was reported in only 8 studies included in the review [16–18, 21, 22, 24, 26, 27].

### Risk of bias within studies

Table 1 presents the risk of bias assessment of the studies included in the review. Four of the included studies [18, 19, 26, 27] were conducted with a sound methodology and therefore, received a maximum of 9 stars according to the scale. Among the remaining eight studies, six [16, 17, 20, 22, 24, 25] received 8 stars and the rest two studies [21, 23] were assigned 7 stars. The lack of control of the confounding factors in the included studies led to receiving fewer stars in the "*comparability*" domain. In the "*selection*" domain, the studies received fewer stars because the selection of the participants was performed from a selective group of users and was not representative of the average in the community.

**Table 1:** Risk of bias assessment (New Castle Ottawa Scale for Cohort studies)

Authors, year	Selection (maximum score = 4^*^)	Comparability (maximum score = 2^*^)	Outcome (maximum score = 3^*^)	Total score (maximum score = 9^*^)
Alaluusua and Renkonen, 1983 [23]	^****^	-	^***^	7^*^
Ansai et al., 2000 [20]	^****^	^*^	^***^	8^*^
Fujiwara et al., 1991 [19]	^****^	^**^	^***^	9^*^
Grindefjord et al., 1995 [26]	^****^	^**^	^***^	9^*^
Litt et al., 1995 [24]	^****^	^*^	^***^	8^*^
Meurman & Pienihäkkinen, 2010 [22]	^****^	^*^	^***^	8^*^
O’Sullivan and Thibodeau, 1996 [25]	^****^	^*^	^***^	8^*^
Seki et al., 2003 [17]	^****^	^*^	^***^	8^*^
Seki et al., 2006 [18]	^****^	^**^	^***^	9^*^
Tenovuo et al., 1990 [21]	^****^	-	^***^	7^*^
Wendt et al., 1996 [16]	^****^	^*^	^***^	8^*^
Xiaoli Gao et al., 2014 [27]	^****^	^**^	^***^	9^*^

### Results of individual studies

Mutans streptococci presence at baseline in the oral samples of preschool children was found to predict early childhood caries in all studies except in 1 study [16] wherein no association was found between the mutans streptococci presence with the development of carious lesions at a later follow-up duration.

### Synthesis of results

**Fig. 2:**
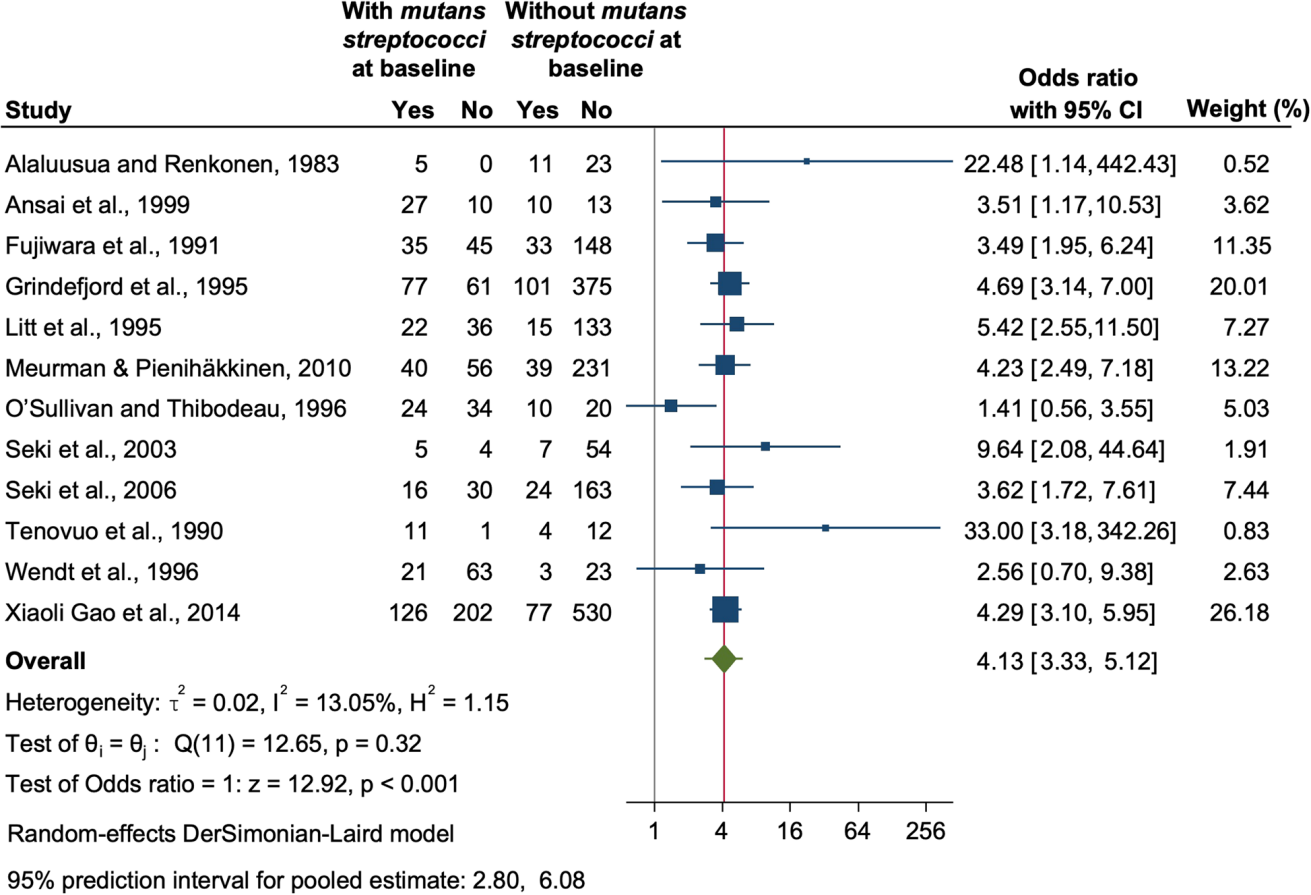
Forest plot of the pooled Odds Ratio (OR) of the development of caries in children with and without mutans streptococci

**Fig. 3:**
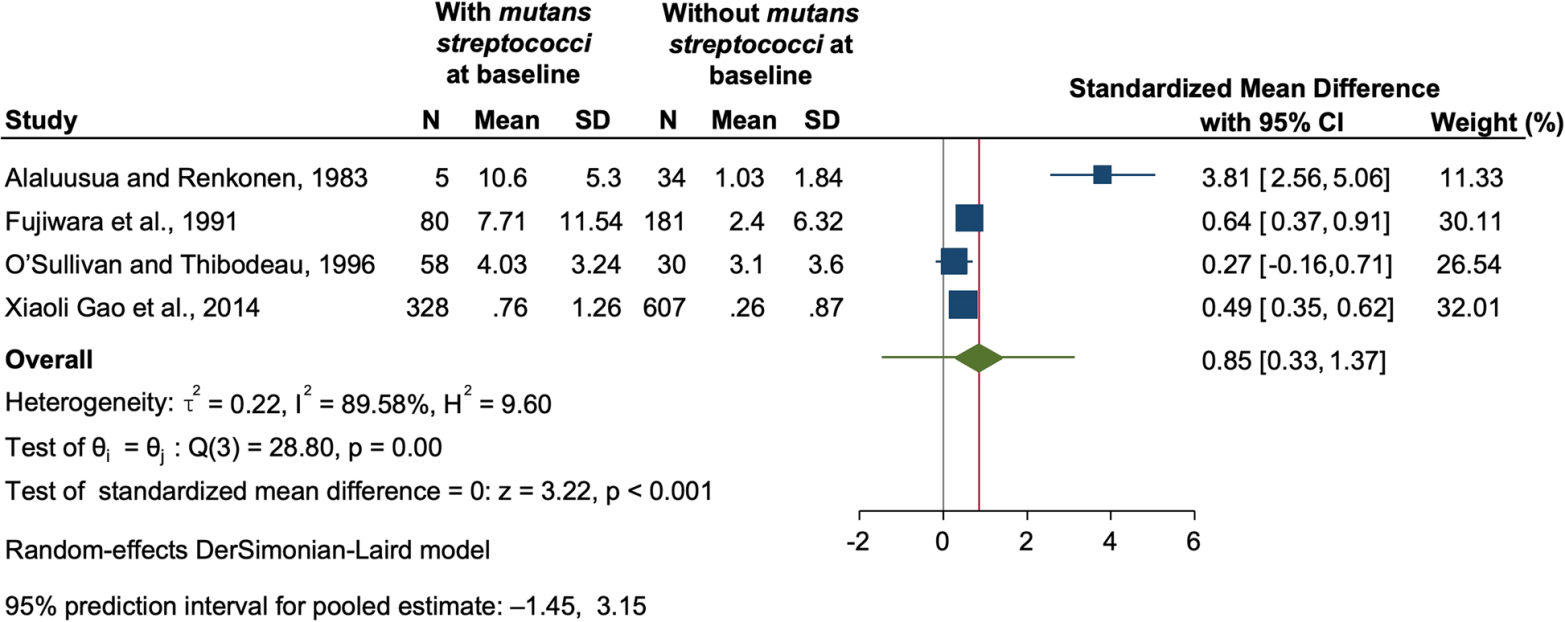
Forest plot of Standardized mean difference (SMD) of future caries experience in children with and without mutans streptococci

All the 12 studies could be pooled to synthesize the results quantitatively as a 2 by 2 table could be constructed. The pooled odds ratio was found to be 4.13 (95% CI: 3.33, 5.12) suggesting that children with mutans streptococci had 4 times higher odds of having future caries (*p* < 0.001) (Fig. 2). The 95% prediction interval for the pooled estimate was 2.80 to 6.08, which indicates that the future value of the odds ratio in similar studies might lie in between this range. The value of heterogeneity was found to be low with an I-squared value of 13.05% and tau-squared value of 0.02, and a p-value of 0.32. Furthermore, we pooled four studies to compare the future caries experience among children in whom mutans streptococci were identified at baseline to those children with no mutans streptococci detected at baseline (Fig. 3). Two of the included studies used dmft [19, 27] whereas the other 2 studies depicted their results using dmfs [23, 25]. Since the caries experience was on different scales, standardized mean difference was used to compare the continuous outcomes among the two groups [31]. The SMD value of 0.2, 0.5, and 0.8 are widely used, corresponding to small, medium, and large effects. The value of SMD was found to be 0.85 (95% CI: 0.33, 1.37) indicating a large effect (*p* < 0.001). However, in this analysis the value of heterogeneity was high (I-squared 89.58%, *p* < 0.001) which might be due to difference in the follow-ups of the studies, methods used for assessing caries or the samples or techniques used for mutans streptococci identification.

### Additional analysis

**Fig. 4:**
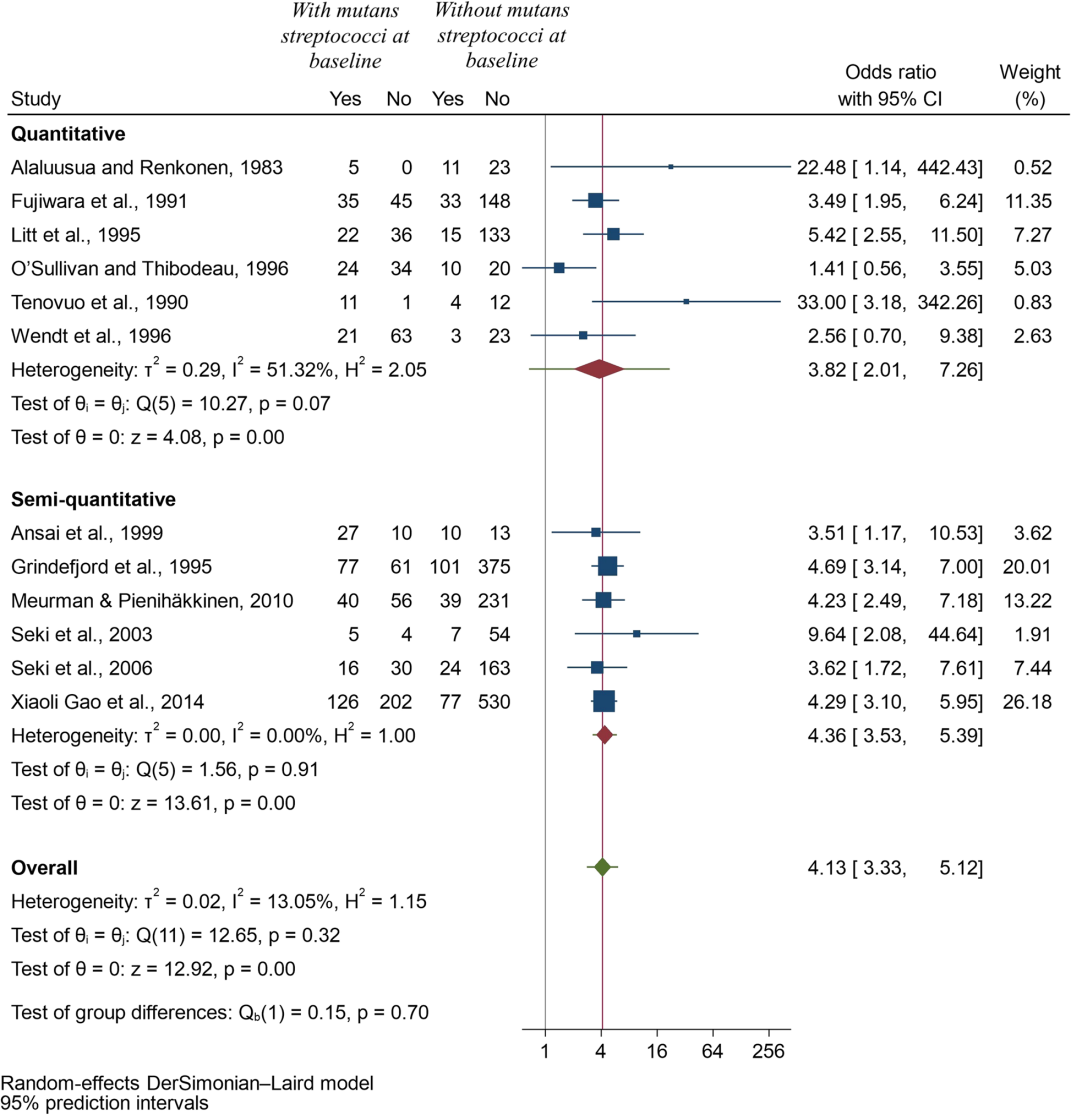
Forest plot of pooled Odds ratio (OR) of development of caries in children with and without mutans streptococci according to method of assessment of mutans streptococci

**Fig. 5:**
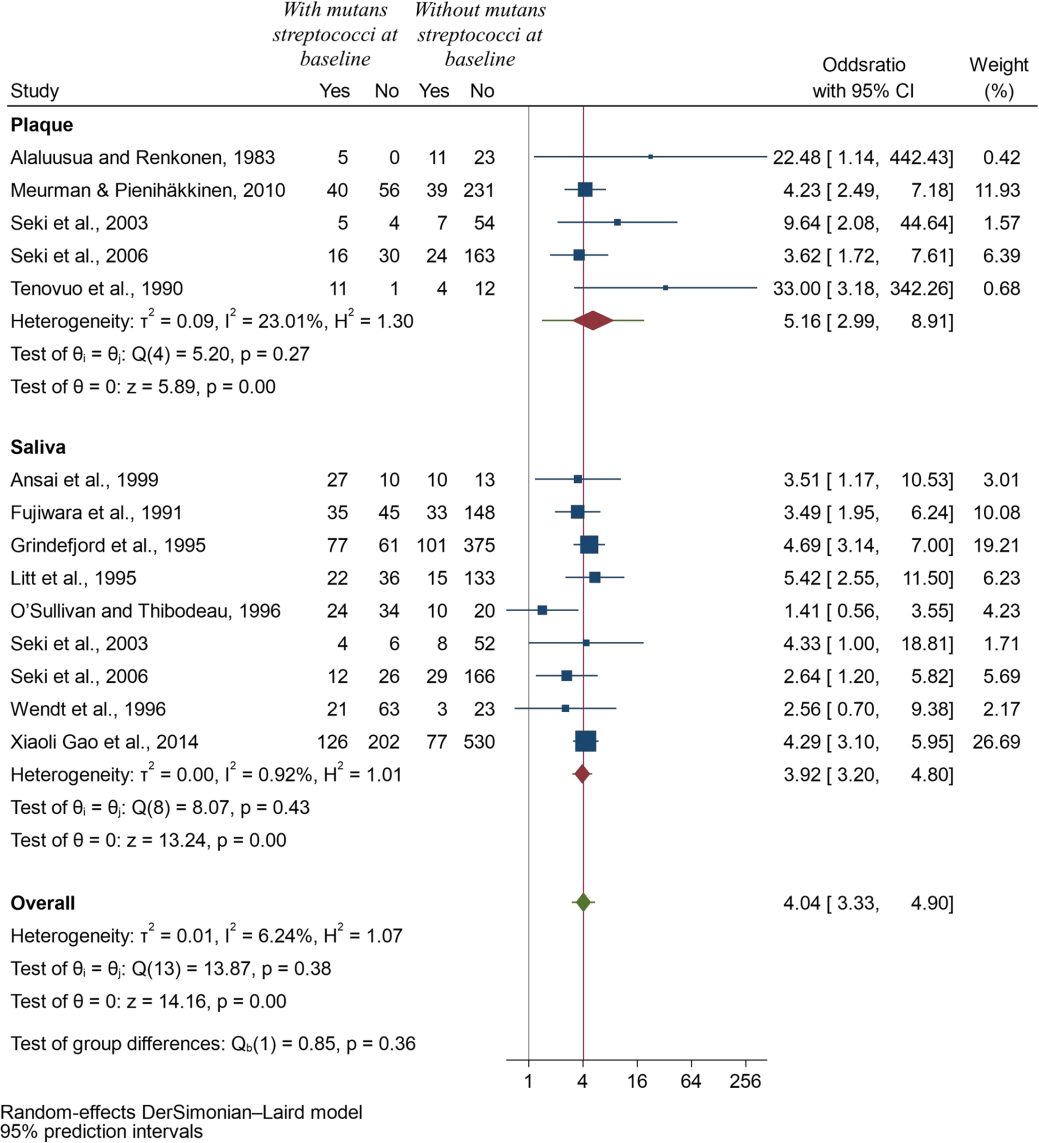
Forest plot of pooled Odds ratio (OR) of development of caries in children with and without mutans streptococci according to the sample used for its detection

**Fig. 6:**
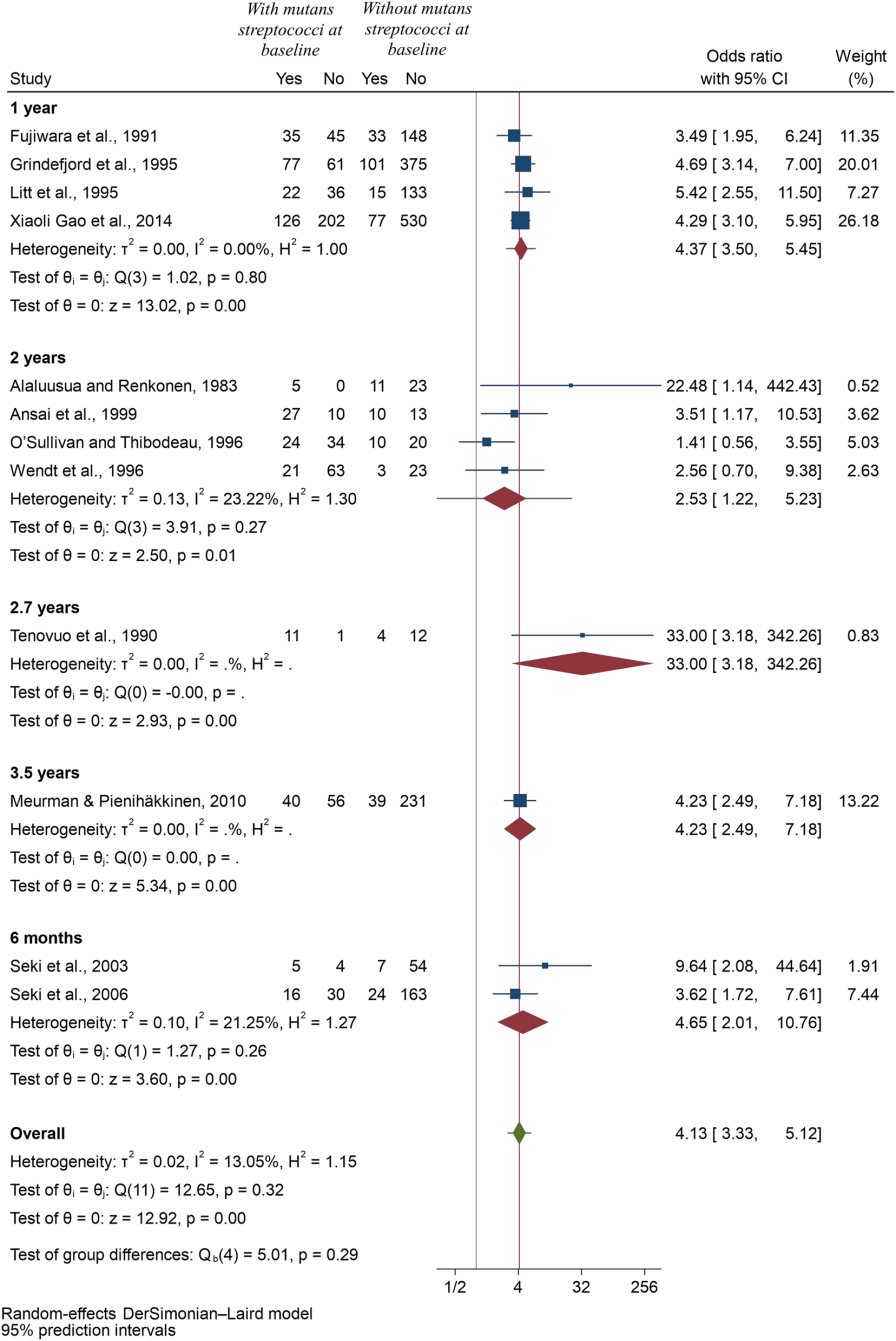
Forest plot of pooled Odds ratio (OR) of development of caries in children with and without mutans streptococci according to the length of follow-ups of the studies

- *Method of assessment of mutans streptococci:* Six studies [16, 19, 21, 23–25] used quantitative methods and remaining six [17, 18, 20, 22, 26, 27] used semi-quantitative methods. The pooled odds ratio for the development of future caries in children with detectable mutans streptococci using quantitative method was 3.82 (95% CI: 2.01, 7.26; *p* < 0.001), whereas using semi-quantitative method, the value was 4.36 (95% CI: 3.53, 5.39; *p* < 0.001), indicating that the results were robust to confirm the risk prediction of early childhood caries in children with detectable mutans streptococci (Fig. 4).
- *Type of sample used:* 5 studies [17, 18, 21–23] used plaque samples to assess mutans streptococci levels and nine [16–20, 24–27] used salivary samples. Two of the studies [17, 18] compared both plaque and salivary samples, and thus were included twice in this meta-analysis. The result still indicated higher odds of developing future caries in preschool children with mutans streptococci using plaque or saliva samples (Fig. 5).
- *Length of follow-up:* The length of the follow-up did not change any of the results and there were higher odds of future caries after 6-months, one year, 2 years, 2.7 years or 3.5 years in children with detectable level of mutans streptococci. In some sub-groups, only one study could be included, but this analysis allowed us to explore the causes of heterogeneity in remaining sub-groups and I-squared value was zero for 1-year follow-up and only 23.22% for two-year follow-up (Fig. 6).

### Certainty of evidence

Table 2 presents the summary of the evidence profile for evaluating certainty. We found a moderate certainty of evidence for mutans streptococci being a risk predictor of the development of carious lesions in preschool children. Since the included studies were observational cohort studies, we started with a very low level of evidence; however, upgraded by one level due to the dose–response relationship between mutans streptococci and dmft/dmfs as evident by large SMD and further upgraded by one level due to large magnitude of effect as seen in the large OR.

**Table 2:** GRADE evidence profile (using GRADEpro GDT): Is mutans streptococci count a risk predictor of Early Childhood Caries?

Certainty assessment						Number of patients with caries		Effect		Notes	Certainty of evidence
**Number of studies**	**Study design**	**Risk of bias**	**Inconsistency**	**Indirectness**	**Imprecision**	**With *S. mutans* at baseline**	**Without *S. mutans* at baseline**	**Relative (95% CI)**	**Absolute (95% CI)**		
12	Cohort studies	Not serious	Not serious	Not serious	Not serious	409/951 (43.0%)	334/2059(16.2%)	OR- 4.13 (3.33 to 5.12)	282 more per 1,000 (from 230 to 336 more)	• Dose–response relationship between Mutans Streptococci and dmft/dmfs as evident by large SMD • Large magnitude of effect as evident by high OR	⨁⨁⨁_O_ Moderate

Abbreviations: *CI* Confidence Interval, *OR* Odds Ratio, *SMD* Standardized Mean Difference

Other considerations: Strong association between presence of mutans streptococci at baseline and future caries in preschool children and all plausible confounding would reduce the demonstrated effect

Levels of Certainty of evidence

High**-**⨁⨁⨁⨁

Moderate- ⨁⨁⨁

Low- ⨁⨁

Very low- ⨁

### Publication bias

**Fig. 7:**
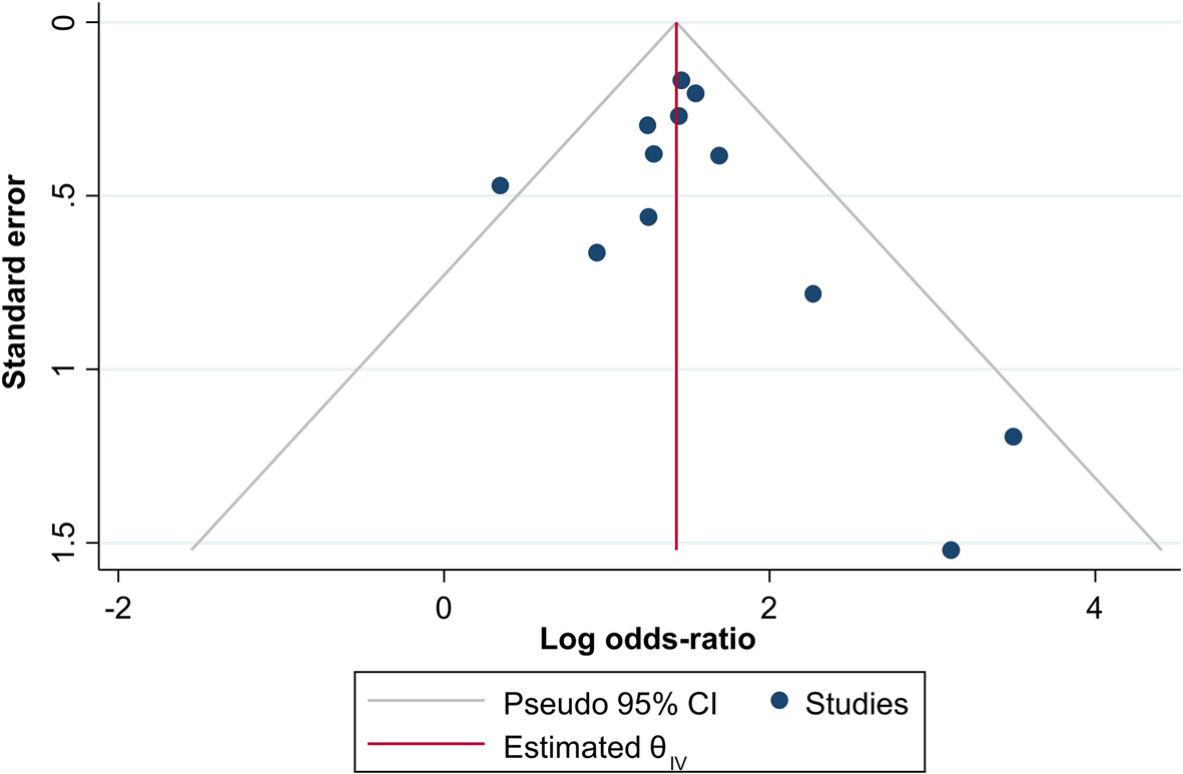
Funnel plot for the publication bias among the included studies

Figure 7 presents the funnel plot generated for checking the publication bias. According to the criteria of symmetry, 11 out of 12 studies included in the plot were inside the funnel, along with being symmetrical and balanced on both sides of the effect size, thus ruling out the publication bias possibility.

## Discussion

The current review found that the presence of mutans streptococci in oral samples of preschool children is a risk predictor for the development of ECC in future. The techniques and the samples used for the microbiological detection of mutans streptococci, along with the study follow-ups varied among the included studies, highlighting the need to cautiously interpret the results.

Half of the included studies have used culturing method for quantification of mutans streptococci, which is considered a gold standard method; however, long time consumption and the need for meticulous growth requirements significantly limit the choice for clinical use [32]. The remaining half of the included studies have used commercially available kits for microbial detection; however, its accuracy, as compared to the advanced laboratory microbiological techniques, is questionable as the latter uses species-specific primers for quantification of a particular bacteria. Furthermore, a previous study [33] has reported a different prevalence of cariogenic bacteria in different intra-oral sites through microbial analysis. The studies included in the current review have collected visible plaque from different sites of the oral cavity: either from the proximal spaces of the maxillary incisors [22] or from the proximal space of deciduous molars and upper incisors [21]. Moreover, the included studies varied in the saliva collection as well because few studies collected unstimulated saliva, while many used stimulated saliva collected after chewing on a paraffin block, which earlier studies have been reported to harbour significantly different microbial profiles [34].

The primary reason for most of the included studies receiving fewer stars in the risk of bias assessment was the lack of control of the various confounding factors. The impact of the confounding factors affecting the outcome of the study has been reported by very few of the included studies. For instance, the reason for the dental visits during the study period may include preventive interventions like fluoride varnish applications, which effects the microbial count of the oral cavity. Previous studies [35–38] have reported the influence of other microbial species like *Bifidobacteria/ S. sobrinus/ Lactobacillus/ Candida* on the mutans streptococci growth in the biofilm. However, not many included studies in the review have quantified other bacteria and evaluated their influence on mutans streptococci colonization. Moreover, the multifactorial nature of ECC demands consideration of other confounding factors like socio-economic status, dietary and oral hygiene factors, parents/caregivers' oral health knowledge and attitude and tooth morphology while evaluating the predictability for ECC development among preschool children. In addition, the majority of the included studies did not select the sample randomly; instead, a convenience sample was used, thus, limiting the generalizability of their results. Therefore, incomplete adjustment for the possible confounding factors might lead to a biased result. Furthermore, the incidence of initial non-cavitated lesions was only assessed by two studies [16, 26] included in the review, which has been found in earlier laboratory studies [39] to correlate with the presence of mutans streptococci in both saliva and plaque samples.

### Strengths and limitations

The strengths of the present review were that though the statistical heterogeneity found among the included studies was low, any heterogeneity found was further analyzed by performing many sub-group analyses. Secondly, the detailed search was done as indicated by a high number of yields obtained. Furthermore, grey literature was searched that yielded one article [16]. Moreover, we set a strict inclusion criterion as we excluded studies with any kind of intervention or with pre-dentate children, along with including only caries-free children to avoid any confounding as the past caries experience has been reported a risk predictor of early childhood caries increment by an earlier systematic review with meta-analysis [40]. Additionally, we assessed GRADE certainty of evidence, providing a "summary of Findings" table. On the contrary, the limitation of the current review was that we included articles published only in English language, although it has been found that the exclusion of studies published in languages other than English have little or no summary effect estimates and hence might not lead to any systematic bias [41].

### Future research and recommendations

Although the review findings indicate the caries risk predictability of mutans streptococci presence in preschool children; however, the differences in the methodology of the studies included cannot be ruled out. Therefore, this review recommends that well-designed studies be conducted in the future to confirm the risk predictability of mutans streptococci as well as other cariogenic microorganisms for the development of caries. This, in turn, might help the clinicians with using the mutans streptococci test as a tool for assessing the caries risk of in a child at an early age and thereby implementing appropriate preventive measures like regular dental check-ups, intensive use of fluorides and prophylactic measures. Moreover, management of ECC should focus on inhibiting early mutans streptococci colonization in a child's life, preventing its transmission from people around the child and implementing early preventive measures. The child being in contact with caregivers at home or classmates at schools/nurseries makes them the possible sources of such transmission. Furthermore, future research may focus on addressing the complete transmission pattern of all cariogenic microorganisms that might increase the risk-predictability of the child having caries later. Thus, the review emphasizes the need to implement early preventive measures by the clinicians for all individuals in contact with the child to delay the acquisition of cariogenic microorganisms.

## Conclusions

The current systematic review and meta-analysis found a moderate certainty of evidence for the presence of mutans streptococci early in preschool children as a risk predictor for the development of caries later in life. The findings might help in the early identification of children with increased risk for having future caries requiring intensive early preventive measures.

### Supplementary Information

**Additional file 1:** **Supplementary Table 1.** The complete search strategy of the electronic databases searched with the yields (number of hits). **Supplementary Table 2.** Reason for exclusion of studies after full text reading.  **Supplementary Table 3.** The summary of the studies included in the review.

## Data Availability

The datasets supporting the conclusions of this article are included within the article and its supplementary file.

## Abbreviations

ECC: Early Childhood Caries
PCR: Polymerase Chain Reaction
dmft: Decayed, missing and filled tooth
dmfs: Decayed, missing and filled surface
WSLs: White spot lesions
SMD: Standardized mean difference
OR: Odds Ratio
WHO: World Health Organization
CFU: Colony forming units
