# Supplementary material for: Is Mutans Streptococci count a risk predictor of Early Childhood Caries? A systematic review and meta-analysis
Source: BMC Oral Health. 2023 Sep 7;23:648. doi: 10.1186/s12903-023-03346-8 (PMC10483843; doi:10.1186/s12903-023-03346-8)
Supplement: Supplementary file 1 — Additional file 1: Supplementary Table 1. The complete search strategy of the electronic databases searched with the yields (number of hits). Supplementary Table 2. Reason for exclusion of studies after full text reading. Supplementary Table 3. The summary of the studies included in the review. [file 12903_2023_3346_MOESM1_ESM.docx]

**Supplementary Table 1:** The complete search strategy of the electronic databases searched with the yields (number of hits)

| **Database** | **Keyword** | **Yield** |
| --- | --- | --- |
| **Ovid MEDLINE(R)** and Epub Ahead of Print, In-Process, In-Data-Review & Other Non-Indexed Citations and Daily 1946 to November 14, 2022 |  | 1765 |
|  | streptococcus mutans.mp. or exp Streptococcus mutans/ | 13092 |
|  | streptococci.mp. or Streptococcus/ | 40140 |
|  | Dental caries.mp. or exp Dental Caries/ | 56405 |
|  | Children.mp. or exp Child/ | 2429577 |
|  | 3 and 4 | 22487 |
|  | early childhood caries.mp. | 1990 |
|  | nursing bottle caries.mp. | 53 |
|  | 5 or 6 or 7 | 22895 |
|  | 1 or 2 | 49937 |
|  | 8 and 9 | 1765 |
| **Embase Classic+Embase**1947 to 2022 November 14 |  | 1950 |
|  | streptococcus mutans.mp. or exp Streptococcus mutans/ | 15555 |
|  | streptococci.mp. or Streptococcus/ | 60517 |
|  | Dental caries.mp. or exp Dental Caries/ | 67055 |
|  | Children.mp. or exp Child/ | 3688500 |
|  | 3 and 4 | 25620 |
|  | early childhood caries.mp. | 2014 |
|  | nursing bottle caries.mp. | 58 |
|  | 5 or 6 or 7 | 25872 |
|  | 1 or 2 | 72768 |
|  | 8 and 9 | 1950 |
| **Scopus** | ( TITLE-ABS-KEY ( streptococci OR streptococcus OR mutans AND streptococci ) AND TITLE-ABS-KEY ( ( dental AND caries AND children ) OR ( early AND childhood AND caries ) OR ( nursing AND bottle AND caries ) ) ) | 2169 |
| **Web of Science** | (ALL=(Streptococci OR streptococcus OR mutans streptococci)) AND ALL=(((child OR children) AND (Dental caries)) OR (early childhood caries) OR (nursing bottle caries)) | 2043 |
| **CINAHL** | ( streptococci OR streptococcus OR mutans streptococci ) AND ( (dental caries in children) OR (early childhood caries) OR (nursing bottle caries) ) | 18 |
| **PubMed** | ("streptococci"[All Fields] OR ("streptococcus"[MeSH Terms] OR "streptococcus"[All Fields]) OR ("mutan"[All Fields] OR "mutans"[All Fields])) AND "streptococci"[All Fields] AND ((("dental health services"[MeSH Terms] OR ("dental"[All Fields] AND "health"[All Fields] AND "services"[All Fields]) OR "dental health services"[All Fields] OR "dental"[All Fields] OR "dentally"[All Fields] OR "dentals"[All Fields]) AND ("carie"[All Fields] OR "dental caries"[MeSH Terms] OR ("dental"[All Fields] AND "caries"[All Fields]) OR "dental caries"[All Fields] OR "caries"[All Fields]) AND ("child"[MeSH Terms] OR "child"[All Fields] OR "children"[All Fields] OR "child s"[All Fields] OR "children s"[All Fields] OR "childrens"[All Fields] OR "childs"[All Fields])) OR ("early"[All Fields] AND ("childhood"[All Fields] OR "childhoods"[All Fields]) AND ("carie"[All Fields] OR "dental caries"[MeSH Terms] OR ("dental"[All Fields] AND "caries"[All Fields]) OR "dental caries"[All Fields] OR "caries"[All Fields])) OR (("nursing"[MeSH Terms] OR "nursing"[All Fields] OR "nursings"[All Fields] OR "nursing"[MeSH Subheading] OR "nursing s"[All Fields]) AND ("bottle"[All Fields] OR "bottle s"[All Fields] OR "bottled"[All Fields] OR "bottles"[All Fields] OR "bottling"[All Fields]) AND ("carie"[All Fields] OR "dental caries"[MeSH Terms] OR ("dental"[All Fields] AND "caries"[All Fields]) OR "dental caries"[All Fields] OR "caries"[All Fields]))) | 781 |

**Supplementary Table 2:** Reason for exclusion of studies after full text reading

| **No.** | **Reference** | **Reason** |
| --- | --- | --- |
| 1. | Mutans streptococci and dental caries in schoolchildren in southern Thailand. Teanpaisan, R.; Kintarak, S.; Chuncharoen, C.; Akkayanont, P. Community Dentistry & Oral Epidemiology 1995;23(5):317-8 1995 | Wrong patient population |
| 2. | Characteristics of Streptococcus mutans genotypes and dental caries in children. Cheon, K.; Moser, S. A.; Wiener, H. W.; Whiddon, J.; Momeni, S. S.; Ruby, J. D.; Cutter, G. R.; Childers, N. K. European Journal of Oral Sciences 2013;121(3 PART1):148-155 | Additional intervention |
| 3. | STREPTOCOCCUS-MUTANS, LACTOBACILLI AND DENTAL-CARIES IN SWEDISH CHILDREN 1984 COMPARED TO 1973 Klock, B.; Krasse, B.  Caries Research 1986;20(2):171-171 | Wrong patient population |
| 4. | A cohort study on the association of early mutans streptococci colonisation and dental decay. Laitala, M.; Alanen, P.; Isokangas, P.; Sderling, E.; Pienihkkinen, K.  Caries Research 2012;46(3):228-233 | Additional intervention |
| 5. | Oral Microbiota Composition Predicts Early Childhood Caries Onset. Grier, A.; Myers, J. A.; O'Connor, T. G.; Quivey, R. G.; Gill, S. R.; Kopycka-Kedzierawski, D. T.  Journal of Dental Research 2021;100(6):599-60 | No specific mutans streptococci detection |
| 6. | Longitudinal clinical and microbiological study on the relationship between infection with Streptococcus mutans and the development of caries in humans. Lang, N. P.; Hotz, P. R.; Gusberti, F. A.; Joss, A. Oral microbiology and immunology 1987;2(1):39-47 | Wrong patient population |
| 7. | Validity of caries risk assessment programmes in preschool children. Gao, X.; Di Wu, I.; Lo, E. C.; Chu, C. H.; Hsu, C. Y.; Wong, M. C. Journal of Dentistry 2013;41(9):787-95 | Wrong study design |
| 8. | A longitudinal study of caries onset in initially caries-free children and baseline salivary mutans streptococci levels: a Kaplan-Meier survival analysis. Kopycka-Kedzierawski, D. T.; Billings, R. J. Community Dentistry & Oral Epidemiology 2004;32(3):201-9 | Wrong patient population |
| 9. | SALIVARY MUTANS STREPTOCOCCI ENUMERATION IN CARIES PREVENTION OF PRESCHOOL-CHILDREN. Twetman, S.; Nederfors, T. Journal of Dental Research 1994;73:415-415 | Additional intervention |
| 10. | A longitudinal controlled study of factors associated with mutans streptococci infection and caries lesion initiation in children 21 to 72 months old. Law, V.; Seow, W. K. Pediatric Dentistry 2006;28(1):58-65 | Additional intervention |
| 11. | Analysis of caries susceptibility factors during transition from caries-free to caries in three-year-old children. Hao, W.; Xu, H.; Chen, X.; Zhou, Q.; Zhang, P.; Qin, M. Chung-Hua Kou Chiang i Hsueh Tsa Chih Chinese Journal of Stomatology 2014;49(4):193-8 | Not in English |
| 12. | Caries risk assessment from dental plaque and salivary Streptococcus mutans counts on two culture media  Sanchez-Perez, L.; Acosta-Gio, A. E. Archives of Oral Biology 2001;46(1):49-55 | Wrong patient population |
| 13. | A Predominant Cariogenic Genotype of Streptococcus mutans in Schoolchildren of Mexico City: A Follow-Up Study. Álvarez-Castro, R.; Bustos-Martínez, J.; Acosta-Gío, A. E.; Hamdan-Partida, A.; Sánchez-Pérez, L. Jundishapur Journal of Microbiology 2019;12(4): | Wrong patient population |
| 14. | Mutans streptococci colonization and longitudinal caries detection with laser fluorescence in fissures of newly erupted 1st permanent molars. Lundberg, P.; Morhed-Hultvall, M. L.; Twetman, S. Acta Odontologica Scandinavica 2007;65(4):189-193 | Wrong patient population |
| 15. | Mutans streptococci and caries development in 1-2.5-year-old children: a longitudinal study. Mattos-Graner, R. O.; Zelante, F.; Correa, Msnp; Mayer, M. P. A. Journal of Dental Research 1998;77:840-840 | Commentary/conference abstract |
| 16. | Oral microbiome development during childhood: an ecological succession influenced by postnatal factors and associated with tooth decay. Dzidic, M.; Collado, M. C.; Abrahamsson, T.; Artacho, A.; Stensson, M.; Jenmalm, M. C.; Mira, A. The ISME journal 2018;12(9):2292-2306 | Additional intervention |
| 17. | MUTANS STREPTOCOCCI AND DENTAL-CARIES PATTERNS IN PRESCHOOL-CHILDREN. Thibodeau, E. A.; Douglass, J. M.; Osullivan, D. M. Journal of Dental Research 1995;74:500-500 | Commentary/conference abstract |
| 18. | Prediction of dental caries development in 1-year-old children. Grindefjord, M.; Dahllof, G.; Nilsson, B.; Modeer, T. Caries research 1995;29(5):343-348 | Children with caries at baseline |
| 19. | Lactobacilli, mutants streptococci and dental caries: a longitudinal study in 2-year-old children up to the age of 5 years. Roeters, F. J.; van der Hoeven, J. S.; Burgersdijk, R. C.; Schaeken, M. J. Caries Research 1995;29(4):272-9 | Children with caries at baseline |
| 20. | Mutans Streptococci and Dental Caries: A New Statistical Modeling Approach. Ghazal, T. S.; Levy, S. M.; Childers, N. K.; Carter, K. D.; Caplan, D. J.; Warren, J. J.; Cavanaugh, J. E.; Kolker, J. Caries Research 2018;52(3):246-252 | Additional intervention |
| 21. | A biopsychosocial model to predict caries in preschool children. Reisine, S.; Litt, M.; Tinanoff, N. Pediatric Dentistry 1994;16(6):413-8 | Wrong study design |
| 22. | The Queensland Birth Cohort Study for Early Childhood Caries: Results at 7 Years. Harrison-Barry, L.; Elsworthy, K.; Pukallus, M.; Leishman, S. J.; Boocock, H.; Walsh, L. J.; Seow, W. K. JDR clinical and translational research 2022;7(1):80-89 | Additional intervention |
| 23. | Delaying Streptococcus mutans Colonization in Children Leads to Reduced Caries Experience. Banas, J. A. Journal of Evidence-Based Dental Practice 2013;13(2):67-69 | Commentary/conference abstract |
| 24. | A Longitudinal Study of Early Childhood Caries and Associated Factors in Brazilian Children. Piva, F.; Pereira, J. T.; Luz, P. B.; Hashizume, L. N.; Hugo, F. N.; Araujo, F. B. Brazilian dental journal 2017;28(2):241-248 | Wrong study design |
| 25. | Establishment and Development of Oral Microflora in 12–24 Month-Old Toddlers Monitored by High-Throughput Sequencing. Li, F.; Tao, D.; Feng, X.; Wong, M. C. M.; Lu, H. Frontiers in Cellular and Infection Microbiology 2018;8 | No specific mutans streptococci detection |
| 26. | Decreasing caries prevalence in Japanese preschool children is accompanied with a reduction in mutans streptococci infection. Seki, M.; Yamashita, Y.  International Dental Journal 2005;55(2):100-4 | Wrong study design |
| 27. | Cariogenic bacteria in a longitudinal study of approximal caries. Sigurjons, H.; Magnusdottir, M. O.; Holbrook, W. P. Caries Research 1995;29(1):42-5 | Wrong patient population |
| 28. | Maturation of the Oral Microbiome in Caries-Free Toddlers: A Longitudinal Study. Kahharova, D.; Brandt, B. W.; Buijs, M. J.; Peters, M.; Jackson, R.; Eckert, G.; Katz, B.; Keels, M. A.; Levy, S. M.; Fontana, M.; Zaura, E. Journal of Dental Research 2020;99(2):159-167 | Wrong outcome |
| 29. | Dental caries and cariogenic factors in pre-school urban Icelandic children. Holbrook, W. P. Caries Research 1993;27(5):431 | Children with caries at baseline |
| 30. | Factors associated with colonization of Streptococcus mutans in 8-to 32-month-old children: A cohort study. Zhou, Y.; Yang, J. Y.; Zhi, Q. H.; Tao, Y.; Qiu, R. M.; Lin, H. C. Australian Dental Journal 2013;58(4):507-513 | Wrong outcome |
| 31. | A multivariate model to predict caries increment in Montreal children aged 5 years. Demers, M.; Brodeur, J. M.; Mouton, C.; Simard, P. L.; Trahan, L.; Veilleux, G.  Community dental health 1992;9(3):273-281 | Children with caries at baseline |
| 32. | Diet may be associated with the detection of cariogenic bacteria in children with early childhood caries. Hague, A. L. Journal of Evidence-Based Dental Practice 2011;11(3):153-155 | Commentary/conference abstract |
| 33. | Experiences with the Streptococcus Mutans in Lakota Sioux (SMILeS) Study: Risk factors for Caries in American Indian Children 0-3 Years. Drake, D.; Dawson, D.; Kramer, K.; Schumacher, A.; Warren, J.; Marshall, T.; Starr, D.; Phipps, K. Journal of Health Disparities Research and Practice 2015;8(3):123-132 | Wrong study design |
| 34. | Assessment of caries risk in preschool children. Pienihakkinen, K.; Jokela, J.; Alanen, P. Caries Research 2004;38(2):156-62 | Additional intervention |
| 35. | Validation of different Cariogram settings and factor combinations in preschool children from areas with high caries risk. Birpou, E.; Agouropoulos, A.; Twetman, S.; Kavvadia, K. International journal of paediatric dentistry 2019;29(4):448-455 | Wrong study design |
| 36. | Social, Psychological, and Behavioral Predictors of Salivary Bacteria, Yeast in Caries-Free Children. Kopycka-Kedzierawski, D. T.; Scott-Anne, K.; Ragusa, P. G.; Cvetanovska, M.; Flint, K.; Feng, C.; Watson, G. E.; Wong, C. L.; Billings, R. J.; Quivey, R. J.; O'Connor, T. G. JDR clinical and translational research 2022;7(2):163-173 | Wrong study design |
| 37. | Dental caries and mutans streptococci levels in preschool children: a community research pilot project  McDonald, H.; Bassett, S.; Hargreaves, J. A.; Williamson, M. F. Probe 1996;30(4):132-5 | Wrong study design |
| 38. | Predicting dental caries increment using salivary biomarkers in a remote Indigenous Australian child population. Fernando, S.; Tadakamadla, S.; Kroon, J.; Lalloo, R.; Johnson, N. W. BMC Oral Health 2021;21(1):37 | Additional intervention |
| 39. | A Predominant Cariogenic Genotype of Streptococcus mutans in Schoolchildren of Mexico City: A Follow-Up Study. Alvarez-Castro, R.; Bustos-Martinez, J.; Acosta-Gio, A. E.; Hamdan-Partida, A.; Sanchez-Perez, L.  Jundishapur Journal of Microbiology 2019;12(4) | Wrong patient population |
| 40. | Relationship of quantitative salivary levels of Streptococcus mutans and S. sobrinus in mothers to caries status and colonization of mutans streptococci in plaque in their 2.5-year-old children. Kishi, M.; Abe, A.; Kishi, K.; Ohara-Nemoto, Y.; Kimura, S.; Yonemitsu, M. Community Dentistry & Oral Epidemiology 2009;37(3):241-9 | Additional intervention |
| 41. | Prospective study of potential sources of Streptococcus mutans transmission in nursery school children. Alves AC, Nogueira RD, Stipp RN, et al. J Med Microbiol. 2009;58(Pt 4):476-481. doi:10.1099/jmm.0.005777-0 | Wrong study design |
| 42. | A long-term effect of caries-related factors in initially caries-free children. Cogulu D, Ersin NK, Uzel A, Eronat N, Aksit S. *Int J Paediatr Dent*. 2008;18(5):361-367. doi:10.1111/j.1365-263x.2007.00859.x | Wrong study design |
| 43. | Dentin caries risk indicators in 1-year-olds. A two year follow-up study. Hultquist AI, Bågesund M. Acta Odontol Scand. 2016;74(8):613-619. doi:10.1080/00016357.2016.1227085 | Wrong study design |
| 44. | A Prospective Longitudinal Study of Early Childhood Caries Onset in Initially Caries-Free Children. Kopycka-Kedzierawski DT, Billings RJ, Feng C, et al. [published online ahead of print, 2022 Jun 9]. JDR Clin Trans Res. 2022;23800844221101800. doi:10.1177/23800844221101800 | Wrong study design |
| 45. | A Two-Year Longitudinal Study of the Effectiveness of the CRT® Bacteria Test in Evaluating Caries Risk in Three-Year-Old Children. Liu Y, Meng Y, Wu M, Zhang Q. Evid Based Complement Alternat Med. 2021;2021:7488855. Published 2021 Oct 5. doi:10.1155/2021/7488855 | Wrong study design |
| 46. | Salivary antimicrobial proteins associate with age-related changes in streptococcal composition in dental plaque. Malcolm J, Sherriff A, Lappin DF, et al. Mol Oral Microbiol. 2014;29(6):284-293. doi:10.1111/omi.12058 | Wrong study design |

**Supplementary Table 3:** The summary of the studies included in the review

| **S. No.** | **Author/Year** | **Follow-up duration** | **Sample size** | **Age** | **Location and Source of funding** | **Caries assessment criteria** | **Sample used** | **Method of count assessment** | **Results** |
| --- | --- | --- | --- | --- | --- | --- | --- | --- | --- |
| 1. 1 | Alaluusua and  Renkonen, 1983 | 2 years | 39/45 | 2 years | Finland, not reported | MOLLER IJ. 1966 | Plaque  and saliva, but data for plaque | Culturing on Mitis  Salivarius bacitracin agar | During the study period, *S. mutans* was detected in 15 of 39 children (38%). At 2, 3 and 4 years of age, 5 (13%), 12 (31%); and 13 (33%) children harboured *S. mutans*, respectively. The children who harbored high levels of *S. mutans* in plaque tended to have it in their saliva as well. Their caries index values (number of decayed, missed and filled surfaces, dmfs = 10.6 + 5.3) at the age of 4 differed significantly from the values of children who harbored *S. mutans* later (dmfs = 3.4+ 1.8, p<0.005) or remained free from *S. mutans* infection (dmfs = 0.3+ 1.1, p<0.0003). |
|  | Ansai et al., 1999 | 2 years | 60 caries-free | Mean age- 2.6 years (Range-0.5 to 6 years) | Japan, not reported | WHO | Stimulated saliva samples | Dentocult Strip  Mutans test (Orion Diagnostica, Espoo, Finland). | Among 23 children, *S. mutans* was not detected at baseline, while 62% were at risk with 15, 13 and 9 considering at low, moderate, and high risk. Cox proportional hazards regression analysis found the relative risk for *S. mutans* as positive, indicating a higher hazard rate of caries development with high *S. mutans* levels compared to low *S. mutans* levels. 90% children with high *S. mutans* level at baseline had caries 9 months later, while only 23% children with not detected level of *S. mutans* at baseline, developed caries at the same period. |
|  | Fujiwara et al.,  1991 | 1 year | 261 caries -free | 0.5 to 1 years | Japan, not reported | WHO | Unstimulated saliva sample | Mitis salivarius  Agar (Difco Laboratories, Detroit, Mi, USA), Colony forming units | 80 children with no caries but with *S. mutans* at baseline, showed a significantly higher caries increment than 181 children who had neither caries nor *S. mutans* at baseline. After 1 year, 33 children from 181 children with *S. mutans* negative at baseline, developed caries. However, 35 children from 80 children with *S. mutans* positive at baseline, developed caries after 1 year. |
|  | Grindefjord et al., 1995 | 1 year | 614 caries-free | 2.5 to 3.5 years (30±3 months) | Sweden, Grants from the Commission for Social Research (Project No. C88/198:2), the Swedish Ministry of Health and Social Affairs, the Swedish Dental Society | Koch criteria, 1967 | Unstimulated saliva from the tongue | Dentocult Strip  Mutans test (Orion Diagnostica, Espoo, Finland). | 29% (178) children manifest caries after 1 year with mean decayed surfaces of 0.9±2.2, with a total of 777 new lesions. 43% of children developing caries after 1 year had *S. mutans* detected at baseline. |
| 1. 13. | Litt et al.,  1995 | 1 year | 184 | 3-4 years | USA, National Institutes of  Health grant No. DE-09217 | Radike 1968 | Unstimulated Saliva | Culturing on Mitis  Salivarius bacitracin agar | Distribution of caries was positively skewed, with most children having few active or treated carious lesions. The distribution of *S. mutans* also was positively skewed, with most children having either no detectable colonies or fewer than 51. The skewness was even more pronounced after 1 year; 73 percent of the children had fewer than 51 *S. mutans* colonies. |
| 1. 4. | Meurman & Pienihäkkinen, 2010 | 42 months | 366/545 | 18 months | Finland,  The Research Fund of the Finnish Dental  Organizations | dmft index | Plaque | Dentocult SM Strip mutans   test (Orion, Espoo,  Finland) kit | At baseline, 1 child (0.2%) was diagnosed with dentinal caries and at the age of 5 years 6 months, in 79 (21.6%) children the 42-month caries increment was >0. In the multivariate regression analysis, the strongest correlation (OR=3.4; 95% CI: 1.9–6.1) was found with *S. mutans* detected in the oral biofilm. |
|  | O’Sullivan and Thibodeau, 1996 | 2 years | 88 caries-free | Mean- 3.8 years | USA, not reported | Radike, 1968 | Unstimulated Saliva | Culturing on Mitis  Salivarius bacitracin agar  Petri dishes | 34, 49 and 17% children had low, moderate, and high SM levels. Children with high *S. mutans* level were least likely to remain caries free, and those who developed caries, nearly 50% had a Δdmfs of more than 4 surfaces (mean dmfs 7.56±4.22). However, 3% and 5% of children with low and moderate *S. mutans* level at baseline, respectively, had a Δdmfs greater than 4 surfaces with mean dmfs as 3.10±3.60 and 2.80±1.52, respectively. Children in the low *S. mutans* group were most likely to remain caries-free and of those who developed caries, majority had an increment of 1 or 2 surfaces. |
| 1. 15. | Seki et al.,  2003 | 6 months | 129 | 3.8 years | Japan, Uemura fund, Nihon  University School of Dentistry, and a grant to  promote multidisciplinary research projects from  the Ministry of Education, Science, Sports, Culture  and Technology of Japan. | WHO | Plaque and saliva | Dentocult SM Strip mutans | The plaque *S. mutans* score was significantly correlated with caries incidence, regardless of caries experience (with and without caries) at baseline (p= 0.001). There was a weak, but significant, relationship between caries incidence and saliva *S. mutans* score when caries experience was absent at baseline (p= 0.038). Caries experience at baseline, plaque *S. mutans* score, and saliva *S. mutans* score to predict caries incidence was examined in a univariate analysis and were significantly associated with caries incidence, with odds ratios 11.01, 15.26 and 5.78, respectively. When these variables were entered in a logistic regression, only two variables remained significant: caries experience at baseline (OR = 5.02, 95%CI: 1.81–14.59) and high plaque *S. mutans* score (2 or 3) (OR = 12.59, 95%CI: 3.18–67.08). |
|  | Seki et al., 2006 | 6 months | 233/410 caries free | 3-4 years | Japan, Sato Fund, Nihon University School of  Dentistry, and a grant to promote multidisciplinary  research projects from the  Ministry of Education, Science, Sports,  Culture and Technology of Japan. | WHO, 1987 | Unstimulated saliva and plaque | Dentocult SM Strip  mutans and Site Strip (Orion Diagnostica,  Espoo, Finland) were used  followed by PCR | There was a significant relationship between caries development and saliva *S. mutans* score when caries experience was absent at baseline (Chi-square test, P = 0.013). Similarly, there was a significant correlation between the plaque *S. mutans* score and caries development (p<0.001) |
| 1. 16. | Tenovuo  et al., 1990 | 2.7 years | 28 caries-free | 0.8 to 3.8 years (median 1.9 years) | Finland,  Supported by Finnish Dental Society and the Academy of Finland. | Dmfs indices | Plaque | Culturing on Mitis  Salivarius bacitracin agar | 43% (12/28) of the children harboured *S. mutans* in their plaque samples. After the follow-up period, 54% (15/28) of these children had carious lesions in their teeth (median dmfs 3.5, range 1-13). In the group of children who developed caries, *S. mutans* levels ranged from 0 to 1,250 x 103 cfu per sample. In the caries-free group, the only *S. mutans* -positive child had 800 cfu of *S. mutans* in her plaque sample. *S. mutans* -positive and -negative groups differed statistically significantly with respect to caries incidence rate. |
| 1. 18. | Wendt et al., 1996 | 2 year | 110  Caries-free | 1 year | Sweden, Swedish Patent  Revenue Fund for Dental Prophylactics, the Swedish Dental Society,  and the Faculty of Odontology, Goteborg University. | Koch criteria, 1967 | Unstimulated Saliva | Culturing on Mitis  Salivarius bacitracin agar | The logistic regression analysis including the microbiologic variables showed no statistically significant association between the presence of *S. mutans* at 1 or at 2 years of age and carious lesions at 3 years of age. |
| 1. 2. | Xiaoli Gao  et al., 2014 | 12 months | 1064 caries-free/1782 | 3-5 years | Singapore, Singapore  Ministry of Education Academic Research Funds R222-000-021-112 and R222-000-022-112. | World Health Organization (WHO) | Simulated Saliva | Dentocult SM Strip (Orion Diagnostica, Espoo, Finland) | At baseline, 1064 (60 percent) children were caries-free. In 12 months, 206 children who were cavity-free at baseline developed new carious lesions (dmft increment Δdmft>0), on at least one originally intact tooth. Compared with children having an *S. mutans* score of 0, those with scores of 1, 2, and 3 were significantly more likely to have new caries, as reflected by an RR (95% CI) of 1.77 (1.29-2.06), 2.00 (1.72-2.16), and 2.19 (2.04-2.25), respectively. Compared with children who were cavity-free at baseline, those found with any cavity at the start of the study (baseline dmft>0) were 1.62 (95% CI=1.25-1.90) times as likely to develop caries in 12 months. Among children who were caries free at baseline, the mean caries increment, percent with new caries, and RR for each stratum was lower vs statistics from children with caries at baseline. |
